# Association of 17-β Estradiol with Adipose-Derived Stem Cells: New Strategy to Produce Functional Myogenic Differentiated Cells with a Nano-Scaffold for Tissue Engineering

**DOI:** 10.1371/journal.pone.0164918

**Published:** 2016-10-26

**Authors:** Chunxiang Feng, Jinqian Hu, Chang Liu, Shiliang Liu, Guiying Liao, Linjie Song, Xiaoyong Zeng

**Affiliations:** 1 Department of Urology, Tongji Hospital, Tongji Medical College, Huazhong University of Science and Technology, Wuhan, Hubei, China; 2 School of Material Science and Chemistry Engineering, China University of Geosciences (Wuhan), Wuhan, Hubei, China; Second University of Naples, ITALY

## Abstract

The increased incidence of stress urinary incontinence (SUI) in postmenopausal women has been proposed to be associated with a reduction in the level of 17-β estradiol (E2). E2 has also been shown to enhance the multi-differentiation ability of adipose-derived stem cells (ASCs) *in vitro*. However, studies on the potential value of E2 for tissue engineering in SUI treatment are rare. In the present study, we successfully fabricated myogenically differentiated ASCs (MD-ASCs), which were seeded onto a Poly(l-lactide)/Poly(e-caprolactone) electrospinning nano-scaffold, and incorporated E2 into the system, with the aim of improving the proliferation and myogenic differentiation of ASCs. ASCs were collected from the inguinal subcutaneous fat of rats. The proliferation and myogenic differentiation of ASCs, as well as the nano-scaffold biocompatibility of MD-ASCs, with or without E2 supplementation, were investigated. We demonstrated that E2 incorporation enhanced the proliferation of ASCs *in vitro*, and the most optimal concentration was 10^−9^ M. E2 also led to modulation of the MD-ASCs phenotype toward a concentrated type with smooth muscle-inductive medium. The expression of early (alpha-smooth muscle actin), mid (calponin), and late-stage (myosin heavy chain) contractile markers in MD-ASCs was enhanced by E2 during the different differentiation stages. Furthermore, the nano-scaffold was biocompatible with MD-ASCs, and cell proliferation was significantly enhanced by E2. Taken together, these results demonstrate that E2 can enhance the proliferation and myogenic differentiation of ASCs and can be used to construct a biocompatible cell/nano-scaffold. These scaffolds with desirable differentiation cells show promising applications for tissue engineering.

## 1. Introduction

The increased incidence of stress urinary incontinence (SUI) in postmenopausal women has been mainly attributed to relaxation of the pelvic floor muscles and a consequent decline in bladder function [1]. In generally, there are two subtypes of SUI: urethral hypermobility caused by anatomic lesions and typically a loss of backstop support at the bladder neck, and intrinsic sphincter deficiency (ISD) due to the weakness of the urethral sphincter [2]. A decrease in estrogen levels is a characteristic and important feature of postmenopausal women, which may be associated with ISD [3]. Adipose-derived stem cells (ASCs) have been considered a promising treatment for ISD to reconstitute the sphincter, because of their multipotentiality, immune privilege, requirement of a low amount of tissue [4–6]. Successful tissue regeneration requires a sufficient cell population with high differentiation capacity. Unfortunately, many factors can reduce the proliferation, population, and differentiation potential of ASCs, including advanced age and the presence of degenerative disease in donors [7, 8]. Although ASCs can be obtained in a sufficient supply with a minimally invasive procedure, their capacity for multiple differentiation is lower than that of bone marrow-derived mesenchymal stem cells (MSCs) [9, 10]. Therefore, effective enhancement of the proliferation and differentiation potential of ASCs is necessary for clinical applications.

The sex steroid estrogen plays multifunctional roles in influencing cell proliferation, differentiation, and metabolism in many tissues. 17-β Estradiol (E2) has been shown to modulate the multiple differentiation capacity of stem cells in the bone, muscle, cartilage, and adipose tissues. In addition, some researchers [11–14] have revealed that E2 can effectively regulate the osteogenesis and adipogenesis of MSCs and significantly improve the osteogenic differentiation potential of MSCs [15]; E2 also plays an important role in regulating muscle function by enhancing the myogenic marker expression of vascular wall-resident stem/progenitor cells or muscle-derived satellite cells (MDSCs) [16]. In animal studies, hypoestrogenism caused by ovariectomy has been shown to induce urothelial atrophy, resulting in a significantly decreased smooth muscle density and increased connective tissue within the detrusor of the urinary bladder [17]. Because estrogen receptors are also located throughout the bladder, urethra, and adipose tissues, we hypothesized that estrogen might also influence the proliferation and myogenic differentiation of ASCs.

In the past few years, ASCs have been used for ISD treatment and have shown functional improvement to the damaged urethra [18, 19]. However, the cells can become injured and experience uncontrollable death during implanting, which negatively affects the therapy outcome [2]. Tissue engineering is a newly emerging strategy that shows potential for the treatment of ISD. The principle of a tissue engineering strategy involves the seeding of cells onto a biomaterial scaffold with or without an active factor for enhancement of cell growth. There are several methods for scaffold fabrication in tissue engineering [20, 21], and electrospinning is one such method that has garnered particular interest. Electrospinning produces fibers at the nanometers to micrometers scale, with a high surface area-to-volume ratio [22]. We here evaluated the use of an electrospinning scaffold with myogenically differentiated ASCs (MD-ASCs) as a feasible new option for SUI treatment.

## 2. Materials and Methods

### 2.1. Isolation and *in vitro* culture of ASCs and smooth muscle cells (SMCs)

Experiments on animals were conducted according to National Institutes of Health Guidelines for the Care and Use of Laboratory Animals and were approved by the Ethics Committee of Tongji Medical College (Permit Number: TJ-A20141214). Inguinal subcutaneous adipose tissue was obtained from 1-month-old Sprague Dawley rats (S1 Table). The rats were sacrificed by cervical dislocation under general anesthesia using 10% chloral hydrate solution (0.4 ml/100 g). Isolation was performed based on our laboratory protocol as described previously [23]. Adipose tissue was washed and minced in a Petri dish with phosphate-buffered saline (PBS), and after removal of the blood vessels, the adipose tissue was dispersed by digestion with 0.1% collagenase type I (Sigma-Aldrich, USA) at 37°C for 120 min with agitation. After one more wash, the layer below the floating lipid-filled adipocytes was dispensed into 15-mL tubes and centrifuged at 1 500 rpm for 10 min. The cells were collected by filtration with a cell strainer with a pore size of 100 mm in diameter. The harvested cells were cultured in low-glucose Dulbecco’s modified Eagle’s medium (LG-DMEM; Gibco, USA), 10% fetal bovine serum (FBS; Gibco, USA), and 100 U/mL penicillin–streptomycin (Boshide, China) at 37°C in 5% CO_2_ at saturating humidity, with medium changes twice weekly. Once the cells reached 70–80% confluence, they were detached with 0.25% trypsin (Sigma-Aldrich) and passaged in 25-cm^2^ flasks at a seeding density of 10^4^ cells/cm^2^. The passage-3 cells were used in the experiments.

SMCs were obtained from the rat urinary bladder in sterile conditions, using enzymatic dispersion technique [24]. In brief, following tissue isolation, the adipose tissue, connective tissue, and adventitia were removed. The tissues were opened and the luminal surface layers were removed mechanically by scraping with a scalpel blade. The tissues were washed three times in PBS containing 100 U/mL penicillin–streptomycin. The tissues were minced and digested for 30 min at 37°C under agitation in PBS containing 15 U/mL elastase (Calbiochem, Germany), 20 U/mL papain (Sigma-Aldrich), and 200 U/mL collagenase type II (Gibco).

### 2.2. Multilineage differentiation and characterization of ASCs

To evaluate their differentiation potential, the third cell-passage ASCs were induced to differentiate into adipocytes and osteoblasts. Briefly, for adipogenic differentiation, the cultured cells were induced with ASCs adipogenic differentiation medium kit (Cyagen Biosciences, China). After 14 days of culture, the cells were fixed in 10% formalin for 10 min and stained with fresh Oil-Red-O solution (Sigma-Aldrich) to visualize the lipid droplets in the induced cells. For osteogenic differentiation, the cultured cells were induced in ASCs osteogenic differentiation medium kit (Cyagen Biosciences, China). After 21 days of culture, cells were confirmed by positive alizarin red (Sigma-Aldrich) staining of mineralized matrix to reveal markers of osteogenic differentiation. Furthermore, ASCs were analyzed for the expression of positive markers (CD90 and CD73, from BD Biosciences, USA) and native markers (CD45, from BD Biosciences, USA) [25]using fluorescence-activated cell sorting (FACS).

### 2.3. *In vitro* proliferation and myogenic differentiation of ASCs with E2 supplementation

The effects of estrogen on cell proliferation were evaluated after ASCs were treated with E2 at concentrations ranging from 10^−7^ to 10^−11^ M. A total of 1 000 cells were placed into each well of a 96-well plate and cultured in steroid-free culture medium (DMEM without FBS) supplemented with E2 (Sigma-Aldrich). The ASCs that grew without E2 supplementation were used as the control group. Cell proliferation was measured using a 3-(4,5-dimethylthiazol-2-yl)-2,5-diphenyltetrazolium bromide (MTT; Beyotime Biotech, China)-based colorimetric method according to the manufacturer’s manual.

To characterize the ASCs after differentiation with E2 supplementation, the cells were exposed to the smooth muscle inductive medium [26], consisting of MCDB131 medium (Sigma-Aldrich), 1% FBS, and 100 units/mL of heparin, supplemented with E2 at 10^−9^ M. The myogenic differentiation of ASCs grown without E2 supplementation served as the control. At week 2 and week 4, the MD-ASCs were collected to examine the expression of SMC-specific markers by quantitative reverse transcription-polymerase chain reaction (RT-PCR) and western blotting analysis, as described below.

Total RNA was isolated from the SMCs, MD-ASCs treated with or without E2, and ASCs, using TRIzol reagent (Invitrogen, Carlsbad, CA, USA), according to the manufacturer instructions. Equal amounts of total RNA were reverse-transcribed into cDNA using ReverTra Ace (Toyobo, Osaka, Japan). The alpha-smooth muscle actin (α-SMA), calponin (Calponin), and myosin heavy chain (MHC) genes were selected for representing the early stage, mid-stage, and late stage of SMC markers, respectively [27]. The primers used were listed in Table 1. RT-PCR was conducted in a 20-μL reaction mixture containing 10 μL of SYBR Green (Toyoba, Japan), 10 pmol of forward primer, 10 pmol of reverse primer, and 1 μg of cDNA. Amplification parameters consisted of an initial denaturation at 95°C for 5 min and 40 cycles of three-step PCR. The data were analyzed using the comparative critical threshold (Ct) method with the amount of the target gene normalized to the average expression level of the control gene (GAPDH). Each experiment was performed at least three times.

**Table 1:** Primer sequences used for quantitative RT-PCR.

Target gene	Primer sequence (5`-3`)
MHC	Forward CCAAGAACATGGACCCGCTAAAT
	Reverse CGGAACATGCCCTTTTTGGTCTT
α-SMA	Forward CAGGGAGTGATGGTTGGAAT
	Reverse GGTGATGATGCCGTGTTCTA
Calponin	Forward AACCCCACGACATTTTTGAG
	Reverse CCCCCACATTGACTTTGTTT
GAPDH	Forward ATGGGTGTGAACCACGAGAA
	Reverse GGCATGGACTGTGGTCATGA

Western blots were performed as described previously [23]. α-SMA and MHC were chosen as markers to verify the differentiation of the cells. Total proteins were extracted and the extraction was scraped into micro-tubes, centrifuged, and stored at -20°C until analysis. Total protein content was determined with the bicinchoninic acid protein reagent. Equal amounts of protein were separated by sodium dodecyl sulfate-polyacrylamide gel electrophoresis and transferred to polyvinylidene difluoride membranes, which were incubated with a primary antibody (all from Abcam, USA) against α-SMA (ab18147) and MHC (ab24642) overnight at 4°C. The membranes were then washed and the secondary antibody (horseradish peroxidase-conjugated anti-mouse IgG antibody; Boster Bio Tech, China) was added. Beta-actin served as an internal control.

### 2.4. Immunocytochemistry

To determine the intracellular expression and localization of muscle contractile proteins in MD-ASCs, immunofluorescent staining was performed. Smooth muscle contractile proteins, including α-SMA and MHC, are specific to differentiated SMCs, consistent with their role in contractile function. MD-ASCs were fixed in 4% paraformaldehyde in PBS for 15 min and then permeabilized with PBS containing 0.1% Triton X-100. For immunostaining, the specimens were incubated with anti-MHC (ab24642) and anti-α-SMA (ab18147) antibody for 2 h, and then with the FITC-conjugated and Cy3-conjugated anti-mouse secondary antibody (Abcam, Cambridge, UK) for 1 h.

### 2.5. Cellar response to the nano-scaffold

#### 2.5.1. Cell morphology

The Poly(l-lactide)/Poly(e-caprolactone) (PLLA/PCL) nano-scaffold that we fabricated in a previous study [22] was used in the present study. In brief, PLLA and PCL were dissolved in hexafluoroisopropanol (HEIP) to a concentration of 10 wt%. The two polymer solutions were blended at a 1/1 ratio, followed by magnetic stirring for 4 h, and added to a 2-mL syringe with a mass flow rate of 1.0 mL/h. The electrospinning was run in an electrical field of 15 kV, and a grounded aluminum foil was used as collector, which was positioned opposite to the needle tip of syringe with a perpendicular distance of 15 cm. To visualize the nano-scaffold under confocal microscopy, a methacrylated rhodamine dye (MeRho, Polysciences) was incorporated prior to electrospinning[28]. All processing steps were performed at room temperature. The nano-scaffold was seeded with MD-ASCs by dropping 0.2 mL of a cell suspension (~30 000 cells), and enabling the cells to attach for 2 h in the incubator at 37°C and 5% CO_2_ before the addition of DMEM with 10% FBS. The cells were then incubated on the scaffold for a further 24 h and fixed using 5% glutaraldehyde. The morphology of MD-ASCs with or without E2 were determined by scanning electron microscopy (SEM) at random locations. For SEM, the constructs were washed in PBS, fixed in 2.5% glutaraldehyde for 20 min, dehydrated in a series of graded concentrations of water/ethanol, air-dried overnight, and gold-sputtered.

#### 2.5.2. Cell attachment

Thirty thousand MD-ASCs grown with or without E2 supplementation were seeded onto the scaffolds and allowed to incubate for 24 h. The scaffolds were encapsulated into a self-manufactured tray, and loaded into a tube filled with cell culture media. The cells/scaffolds were placed in a centrifuge at 57 *g* or 514 *g* for 15 min [29]. The MTT assay was then performed to assess the quantity of cells remaining on the scaffold. Cell adhesion on the electrospun scaffolds after centrifugation was normalized to the MTT absorbance values (at 570 nm) for the same type of cells that had not undergone centrifugation.

#### 2.5.3. Cell proliferation

Thirty thousand MD-ASCs were seeded onto the scaffold, and the proliferation of cells was analyzed on cell culture plates from 1 to 7 d after initial cell seeding. The plates were divided into two groups according to growth in cell culture media with or without E2, E2(+) and E2(-), respectively, and proliferation was assessed from the results of the MTT assay. The cell culture media were changed every 2 d. Five samples for each cells/scaffold group were analyzed for each time point.

#### 2.5.4. Cell differentiation

An immunofluorescence assay was performed to detect α-SMA and MHC expression in ASCs after differentiation with or without E2 supplementation on the scaffolds for 2 and 4 weeks. In brief, the samples were fixed, permeabilized and blocked with 5% bovine serum albumin in PBS. Subsequently, the samples were immersed in anti-MHC (ab24642) and anti-α-SMA (ab18147) antibody overnight at 4°C. After washing with PBS, the samples were incubated with FITC-conjugated anti-mouse secondary antibody (Abcam, Cambridge, UK) at 37°C for 1 h. Finally, the nuclei were counterstained with Hoechst 33258.

### 2.6. Statistical analysis

All experiments were carried out at least three times, and quantitative data are expressed as the mean ± standard deviation. The statistical significance of differences between two groups was assessed by a one-sample t-test. P-values less than 0.05 were considered to indicate a statistically significant difference. All statistical analyses were carried out using SPSS software (SPSS Inc., Chicago, IL, USA).

## 3. Results

### 3.1. Multilineage differentiation and characterization of ASCs

**Fig 1:**
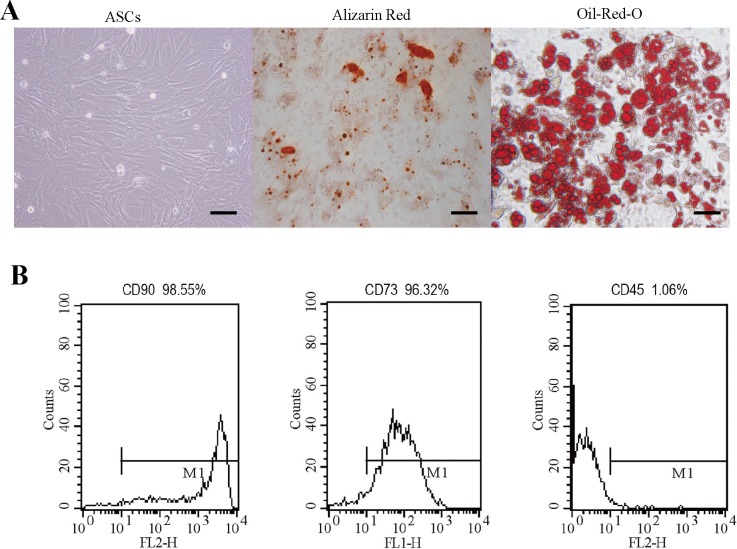
Multilineage differentiation and characterization of ASCs. (A) Microscope images of undifferentiated ASCs at passage 3; Alizarin Red-stained osteogenic ASCs and Oil-O-Red-stained adipocytes differentiated into ASCs. Scale bar = 50 μm. (B) Flow cytometric analysis of ASCs. The ASCs were analyzed by flow cytometry with fluorochrome-conjugated monoclonal antibodies against CD90, CD73s, and CD45.

The passage-3 ASCs showed a polygonal fibroblast-like morphology, with abundant cytoplasm and large nuclei, corresponding with the typical shape of MSCs (Fig 1A). To investigate the adipogenic differentiation potential of the ASCs, the cells were stained with Oil-Red-O to demonstrate the intracellular accumulation of lipid droplets (Fig 1A). The osteogenic differentiation of ASCs was confirmed according to the positive expression of alkaline phosphatase, and the alizarin red stain further indicated the deposition of calcified extracellular matrix (Fig 1A). The ASCs were further characterized by FACS analysis with monoclonal antibodies against CD90, CD73, and CD45, with an expression rate of 98.55%, 96.32%, and 1.06%, respectively, which is in agreement with the typical characterization of ASCs (Fig 1B).

### 3.2. Effect of E2 on ASCs proliferation *in vitro*

**Fig 2:**
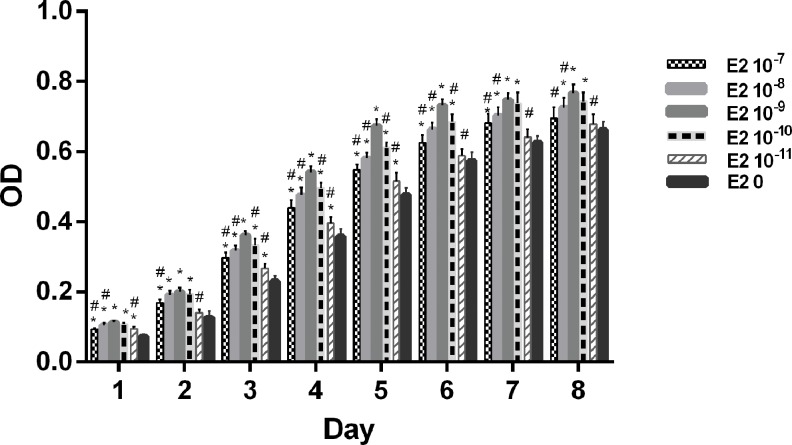
Effect of E2 treatment on ASCs proliferation *in vitro*. ASCs proliferation (OD at 490 nm) after E2 treatment at different concentrations from 1 to 8 days. Student’s t-tests were used to evaluate the significance (*p* < 0.05) in the OD values of cells treated with different concentrations of E2 with those of the control groups (E2-free)(*) and the 10^−9^ M E2 group (#).

Fig 2 summarizes the effects of E2 supplementation in steroid-free culture medium on cell proliferation of ASCs treated at a concentration of 10^−7^–10^-11^M from 1 to 8 d. Treatment of E2 at concentrations of 10^−7^–10^−10^ M significantly increased cell proliferation after 1 to 7 d, whereas 10^−8^–10^−10^ M of E2 and 10^−11^ M of E2 only had an effect on cell proliferation after 8 d and at 1 and 3 to 5 days, respectively (p < 0.05). E2 more effectively induced cell proliferation at a concentration of 10^−9^ M than at the other concentrations tested, such as 10^−7^ M and 10^−11^ M from 1 to 8 d, 10^−8^ M at 1 and 3 to 8 days, and 10^−10^ M from 3 to 6 days (p < 0.05). A more limited range of E2 concentrations was observed to significantly increase cell proliferation in male ASCs (10^−7^–10^−10^ M) than in female ASCs (10^−7^–10^−11^ M) (S1 Fig and S2 Fig).

### 3.3. *In vitro* culture and the myogenic differentiation of ASCs

**Fig 3:**
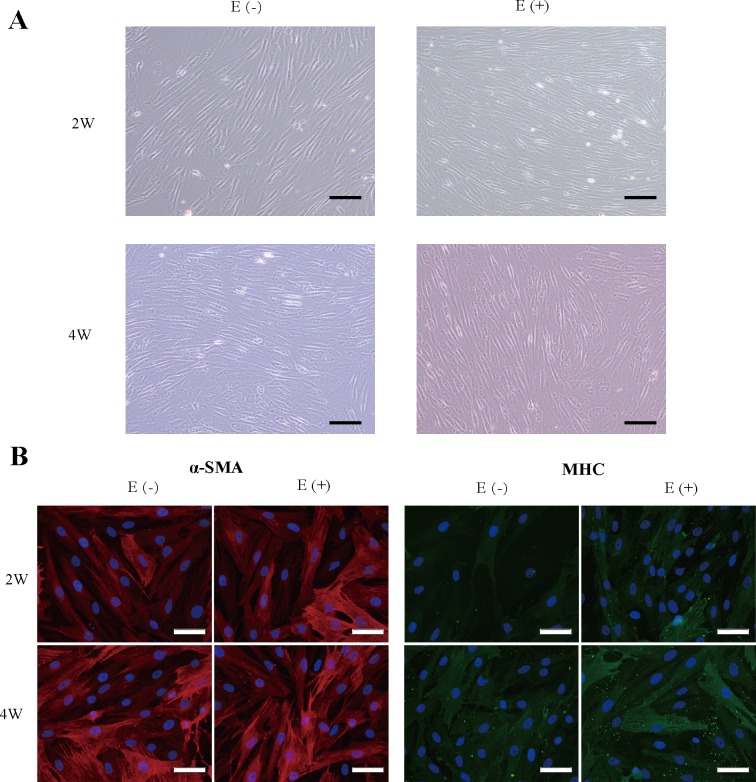
Myogenic differentiation characterization of ASCs with or without E2 supplementation. (A) Phase-contrast images of MD-ASCs treated with and without E2 supplementation for 2 and 4 weeks. Scale bar = 100 μm. (B) Cells were immunostained with antibodies directed against α-SMA (red) and MHC (green); the nuclei are counterstained with DAPI (blue). Scale bar = 50 μm.

After maturation of ASCs with the basic culture medium MCDB131 with or without E2 supplementation for 2 and 4 weeks, the MD-ASCs showed a typical SMC morphology with a spindle-like shape and a ‘‘peak and valley” morphology (Fig 3A). We used Cy3 and FITC conjugated secondary antibody to label the contractile filament network of the 4-week-matured cells to demonstrate whether MD-ASCs could be matured into a contractile phenotype with α-SMA and MHC proteins filaments. MD-ASCs, with or without E2, showed remarkably organized contractile protein fibers; MHC protein was detected expression in the majority of cells, resulting in a fine reticular pattern in the cytoplasm; and α-SMA-positive expression with well-organized, thin SMA filaments (Fig 3B). However, the α-SMA expression in the filaments showed stronger expression than MHC in the cytoplasm.

#### 3.4. Effect of E2 on the expression of myogenic-specific markers

**Fig 4:**
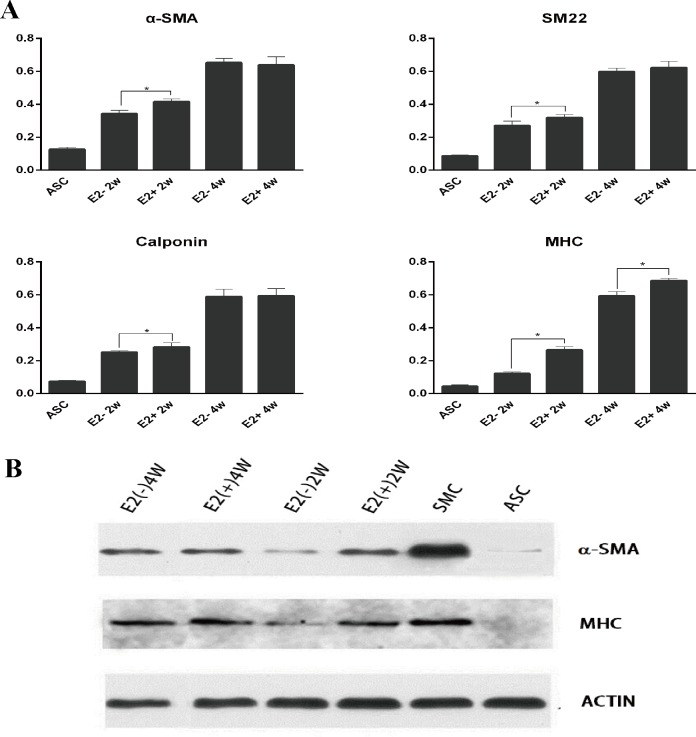
Myogenic differentiation marker expression of ASCs with or without E2 supplementation. (A) Relative expression of myogenic differentiation markers (relative to GAPDH) was measured by quantitative RT–PCR during the myogenic differentiation of ASCs with or without exposure to E2 for 2 or 4 weeks. (B) Western blotting results of the myogenic differentiation of ASCs with or without exposure to E2 for 2 or 4 weeks.

We evaluated the expression of SMC-specific genes of the third-passage MD-ASCs using quantitative RT-PCR. The expression levels of MHC, α-SMA, calponin, and GAPDH were measured by the comparative Ct method (Fig 4A). The Ct values of the genes of the four groups were standardized by the Ct values of the SMCs used as reference. All of the contractile genes were detected in the 2-week-differentiated cells, and especially higher expression was detected in the E2(+) group. After maturation for 4 weeks, α-SMA, calponin, and MHC transcription was upregulated compared to those measured at 2 weeks; consequently, α-SMA and calponin showed similar levels between the two groups, but MHC still showed higher expression in the E2(+) group after 4 weeks of maturation. The same result was detected by western blotting (Fig 4B). E2(-) cells expressed lower levels of α-SMA and MHC protein compared to E2(+) cells after 2 weeks; consequently, these cells showed similar levels of α-SMA but lower expression levels of MHC after 4 weeks of maturation. ASCs expressed a low level of α-SMA; however, MHC expression, as the most restricted marker of SMCs, was not observed. Our results demonstrated that after exposure to myogenic differentiation media supplemented with E2, the ASCs showed improved myogenic capabilities.

### 3.5. Cellular responses to the nano-scaffold

#### 3.5.1. Cells–scaffold morphology

**Fig 5:**
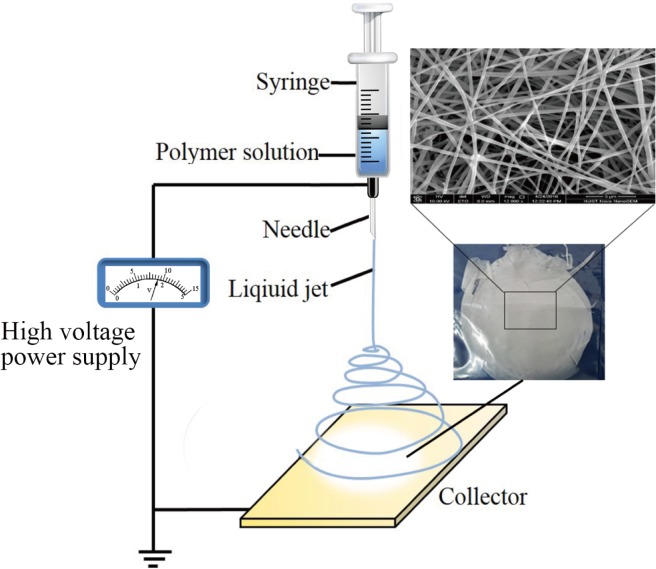
Schematic of an electrospinning system for the production of a nano-scaffold from a polymer solution.

**Fig 6:**
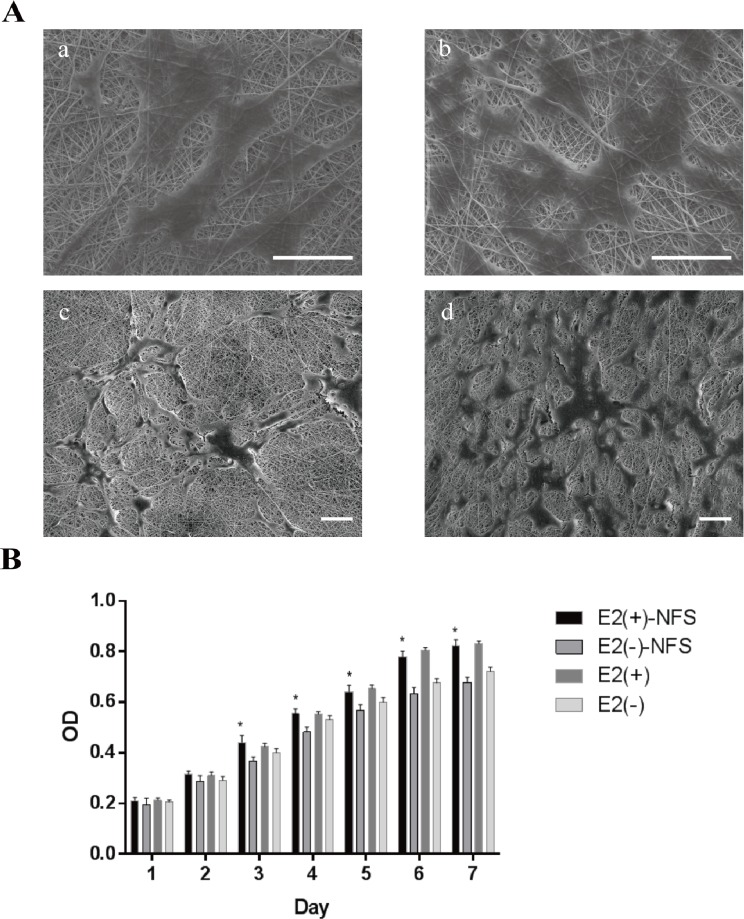
Cellular response to the nano-scaffold. (A) Scanning electron microscopy (SEM) of the morphology of MD-ASCs after 24 h of incubation on the nano-scaffold. (a and b) MD-ASCs treated without E2. (c and d) MD-ASCs treated with E2. Scale bar = 50 μm. (B) Cellular proliferation on a nano-scaffold with or without E2 supplementation. Student’s *t*-tests were used to compare the OD values between groups with and without E2 supplementation (* indicates significance *p <* 0.05).

A schematic of an electrospinning system was shown in Fig 5. At 24 h after seeding the MD-ASCs, we imaged the cell–nano-scaffold complex by SEM. Both groups of cells could be cultured on the nano-scaffold. Cells of the E2(-) group formed small discrete islands (Fig 6A a and c), whereas the E2(+) group showed better cell proliferation to form a cell layer, which was supported by the increase in the spread and density of the cell layer (Fig 6A b and d).

#### 3.5.2. Cellular adhesion

The percentage of MD-ASCs remaining in the scaffold was 82.3 ± 4.1% and 51.7 ± 5.8% after centrifugation at 57 *g* and 514 *g*, respectively. There was no significant difference between the E2(+) and E2(-) groups. Compared to the results of our previous study [22], in which approximately 78.4 ± 4.9% of the cells remained, the MD-ASCs showed sufficient attachment ability to the nano-scaffold.

#### 3.5.3. Cellular proliferation

Cellular proliferation was indirectly determined by measuring the absorbance value of MTT. MD-ASCs seeded on top of the nano-scaffold at the same density showed higher proliferation in the E2(+) culture group than in the E2(-) group during 3 to 7 d of culture (*p* < 0.05) (Fig 6B).

#### 3.5.4. Cellular differentiation

**Fig 7:**
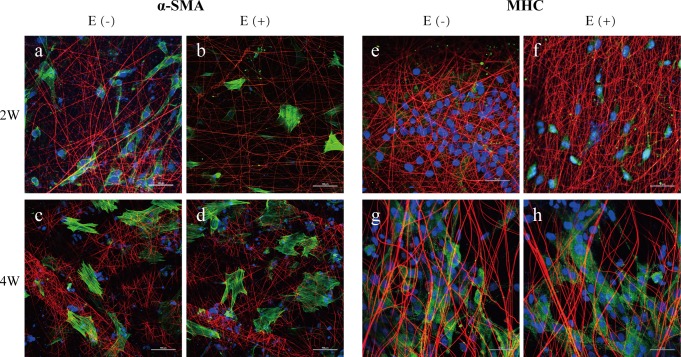
Myogenic differentiation for ASCs on nano-scaffolds with or without E2 supplementation. (a-d) Cells were immunostained with antibodies directed against α-SMA (green) and nano-scaffold fiber (red); (e-h) Cells were immunostained with antibodies directed against MHC (green) and nano-scaffold fiber (red); the nuclei are counterstained with DAPI (blue).Scale bar = 50 μm.

To further investigate the differentiation capacity of stem cells loaded on the nano-scaffold, immunofluorescent staining for α-SMA and MHC was performed at 2 and 4 weeks of myogenic maturation with or without E2 supplementation. After maturation for 2 weeks, α-SMA and MHC showed significantly higher expression in the E2(+) nano-scaffold group (Fig 7A, 7B, 7E and 7F). Subsequently, α-SMA showed similar levels between the two groups (Fig 7C and 7D), but MHC still showed higher expression in the E2(+) nano-scaffold group at 4 weeks (Fig 7G and 7H).

## 4. Discussion

In the present study, we provide direct evidence that E2 stimulates the proliferation and myogenic differentiation of rat ASCs. Our data also demonstrate that the MD-ASCs could proliferate well and with high differentiation potential onto a PLLA/PCL nano-scaffold, which shows potential for clinical treatment.

The choice of appropriate cells with suitable function and cyto-type is essential for a good outcome in tissue engineering. ASCs have multi-directional differentiation potential, and can be induced into SMCs that may be applied to the treatment of muscular organ damage [4, 5, 20]. The reconstruction of smooth muscle requires a large number of high-quality cell sources for good proliferation and differentiation in a short time. However, the current method for inducing ASCs to differentiate into SMCs is a long process [30]; thus, an improved method for the proliferation and differentiation of ASCs is necessary. Although the mechanism remains elusive, the enhancement of E2 may effectively improve applications of ASCs in tissue engineering and regenerative medicine [15, 31, 32].

Previous studies have demonstrated the effects of E2 on increasing the proliferation of MSCs [31, 33]. Our results show that supplementation of E2 in the culture medium can increase the proliferative activity of ASCs at a concentration of 10^−7^–10^−10^ M for cells obtained from male rats and at 10^−7^–10^−11^ M for cells obtained from female rats. These results are in line with those reported previously [31]. The reason for the different optimal E2 concentrations in different sexes may be due to differences in the E2 receptors of ASCs in male and female rats, and the interaction with other steroid hormone receptors.

E2-related receptor-alpha plays a positive role in osteoblastic and adipocytic differentiation [34, 35]. However, the effects of E2 on myogenic differentiation remain controversial. In addition to the impact of gender-specific differences and hormonal levels on cellular mechanisms, the estrogen receptor subtypes and the different conditions of subtype-selective ligands will also have distinct physiological functions at the cellular and molecular levels. Previous research has demonstrated that E2 upregulated MHC-I and downregulated MHC-IIb expression in MDSCs [36]. Another study revealed that the ER/PI3K/Akt/SSH1L axis, but not MAPK signaling, is potentially responsible for the effects of estrogen on MSCs [37]. These findings suggest that the processes involve different mechanisms, which should be elucidated in further studies.

The ultimate goal of tissue engineering is to reconstruct living tissues for the replacement of damaged organs. This requires the use of cells that show sufficient differentiation ability and appropriate function. Thus, the development of a method to differentiate ASCs into matured SMCs should have the following features: 1) conformance with the SMC lineage, and 2) differentiation into a contractile phenotype from an early immature form to a later mature form. In addition, the assembly of α-SMA or MHC protein into filaments serves as a marker of complete differentiation[38, 39]. Our findings suggest that ASCs that were cultured to differentiate into SMCs with or without E2 induced the assembly of MHC and α-SMA protein into organized filaments

α-SMA is an early marker of myocytes, which was first discovered as a differentiated SMC marker during angiogenesis. α-SMA is one of the most abundant actin isoforms in fully differentiated SMCs. However, α-SMA expression alone does not provide definitive evidence for a smooth muscle lineage, owing to the wide variation of non-SMC cell types under certain circumstances [40]. The results of our study showed that expression of α-SMA could be detected in some resting ASCs, and that the expression was upregulated in differentiated ASCs. As the most widely used SMC marker, we consider that the upregulation of α-SMA is useful for representing the relative state of the differentiation and maturation of ASCs. MHC is a marker of the differentiated phenotype, and its expression is restricted to functional smooth muscle and is not detected in any other cell type [30]. Our observation supports the specificity of this marker, as MHC expression was absent in ASCs but was expressed in the differentiated cells, with higher expression observed with a longer maturation period. The expression of α-SMA and MHC appears in chronological sequence during SMCs differentiation. In general, α-SMA is expressed at 2 weeks, followed by the expression of MHC at 4 weeks; subsequently, α-SMA disappears and MHC expression becomes more evident at 12 weeks [30].

Our study observed that the E2 could enhance the expression levels of α-SMA and MHC at 2 weeks of maturation. These effects of α-SMA receded with a longer differentiation maturing time and vanished at 4 weeks, but the level of MHC was still higher at 4 weeks. E2 plays multifunctional roles in influencing differentiation, growth, and metabolism in many tissues. The influence of E2 in the differentiation of cells into other lineages has been previously demonstrated. Specifically, E2 was found to improve the osteogenic and adipogenic differentiation of MSCs [15]. To date, there is no explicit conclusion of the pathways and underlying mechanisms involved in E2 promoting effect on myogenic differentiation, however recent studies showed that E2 upregulated the MHC-I expression of MDSCs [36] and SRC3, an E2 receptor coactivator, could enhance its transcriptional activity in vascular SMCs[41]. In animal studies, a low E2 level caused by ovariectomy was shown to induce urothelial atrophy [42], resulting in significantly increased connective tissue within the detrusor and a decreased smooth muscle density of the urinary bladder [43–45]. The *in vitro* period of cell proliferation and differentiation should be kept as short as possible to allow the organism to act as a bioreactor for further reconstruction. Therefore, the accelerated expression of SMC-specific markers by E2 is a significant finding for improving the outcome of tissue engineering. The National Health and Nutrition Examination Survey showed that nearly 25% of women older than 20 years suffered from SUI, and more than half of these cases were caused by ISD [46]. Implantation of a polypropylene mesh for pelvic floor reconstruction has been widely used in clinical setting; however, the major drawback of tissue erosion remains a problem [47]. Injection of ASCs has shown good potential for curing ISD [18]. Unfortunately, the amount of transplanted cells that survived and participated in tissue repair was relatively low, and thus the efficacy of MSC-based therapy appeared to be marginal. Electrospinning is a promising method for obtaining a non-woven, nanometer-scale structure that can be used as a tissue scaffold. Electrospinning produces fibers on the scale of nanometers with a higher surface to support cell attachment and proliferation [48, 49]. In our previous work, we demonstrated that the nano-fiber scaffold applied in the present study has good biocompatibility. Here, we demonstrated that ASCs can be differentiated into mature SMCs, and show high proliferation on the nano-fiber scaffold with E2 supplementation.

Mature SMCs are characterized as showing either a proliferative “synthetic” phenotype or a biomechanically active, but quiescent, “contractile” phenotype [50]. Such phenotype switching between the two forms could occur reversibly in response to changes in the microenvironmental niche or to mechanical cues [51]. The use of SMCs as part of a tissue engineering strategy for functional tissue replacement requires the reliable expansion of cells while simplifying the processes of dedifferentiation or controlling the processes of cellular redifferentiation towards the desired phenotype [50]. To control the phenotype of SMCs, we used the basic culture medium MCDB131 (1% FBS) and the complete culture medium DMEM (10% FBS). We found that E2 has a dual effect on SMCs differentiation in the different media. In the basic medium, E2 could enhance the expression of SMC-specific markers within 2 weeks, and could stimulate the proliferation of ASC-SMCs in compete medium. These conclusions are similar to the results of previous studies showing that E2 has dual effects on vascular wall-resident stem/progenitor cells. E2 was found to promote the proliferation of undifferentiated stem/progenitor cells by enhancing the binding of the pELK1-SRF complex to the c-Fos gene, whereas it accelerated the myogenic differentiation of differentiating stem/progenitor cells through an SRC3-mediated mechanism [37].

In summary, we have presented a novel method for improving the proliferation and myogenic differentiation of ASCs, which can be combined with a nano-scaffold for tissue engineering applications. However, the pathways involved and the underlying mechanisms of these effects have not yet been confirmed. Further research is required to elucidate the precise mechanisms driving the regulation of estrogen on the myogenic differentiation of ASCs and the associated pathways. Establishment of an animal model of ISD is also needed to evaluate the efficiency of novel E2 nano-scaffold treatments. Nevertheless, this study further enhances the understanding of the functions of estrogens in different cells and provides a novel method for tissue engineering.

## Supporting Information

S1 Fig Effect of E2 treatment on male ASCs proliferation in vitro. ASCs proliferation (OD at 490 nm) after E2 treatment at different concentrations from 1 to 8 days. Student’s t-tests were used to evaluate the significance (p < 0.05) in the OD values of cells treated with different concentrations of E2 with those of the control groups (E2-free)(*) and the 10–9 M E2 group (#).
(TIF)

S2 Fig Effect of E2 treatment on female ASCs proliferation in vitro. ASCs proliferation (OD at 490 nm) after E2 treatment at different concentrations from 1 to 8 days. Student’s t-tests were used to evaluate the significance (p < 0.05) in the OD values of cells treated with different concentrations of E2 with those of the control groups (E2-free)(*) and the 10–9 M E2 group (#).
(TIF)

S1 File Supplementary materials and methods. (DOCX)

S1 Table Characteristics of rats. (DOCX)
