# Supplementary material for: Association of 17-β Estradiol with Adipose-Derived Stem Cells: New Strategy to Produce Functional Myogenic Differentiated Cells with a Nano-Scaffold for Tissue Engineering
Source: PLoS One. 2016 Oct 26;11(10):e0164918. doi: 10.1371/journal.pone.0164918 (PMC5081199; doi:10.1371/journal.pone.0164918)
Supplement: S1 File — (DOCX) [file pone.0164918.s003.docx]

**Supplementary materials and data:**

1. **Ethics:**

We collected and cultured primary ASCs and SMCs from Inguinal subcutaneous adipose tissue and bladder respectively of 8 1-month-old Sprague Dawley rats. Detailed information of donors is provided in Supplementary Table 1. Ethical approval was obtained from the Ethics Committee of Tongji Medical College for this procedure (TJ-A20141214).

All animal procedures were performed in strict accordance with the recommendations in the Guide for the Care and Use of Laboratory Animals of the National Institutes of Health

The rats (SD rats, n = 8) were sacrificed by cervical dislocation under general anesthesia using 10% chloral hydrate solution (0.4 ml/100 g).

The animals were housed according to National Institutes of Health Guidelines for the Care of Laboratory Animals, and had free access to food and water.

2. The effects of estrogen on cell from female or male rats proliferation were evaluated after ASCs were treated with E2 at concentrations ranging from 10^-7^ to 10^-11^ M. A total of 1 000 cells were placed into each well of a 96-well plate and cultured in steroid-free culture medium supplemented with E2. The ASCs that grew without E2 supplementation were used as the control group. Cell proliferation was measured using MTT.

We found that the range of E2 concentrations was more limited to significantly increase cell proliferation in male ASCs (10^-7^–10^-10^ M) (Sup. Fig. 1) than in female ASCs (10^-7^–10^-11^ M) (Sup. Fig. 2). Most optimal concentration was 10^-9^ M in both group.
