# Supplementary material for: Association of 17-β Estradiol with Adipose-Derived Stem Cells: New Strategy to Produce Functional Myogenic Differentiated Cells with a Nano-Scaffold for Tissue Engineering
Source: PLoS One. 2016 Oct 26;11(10):e0164918. doi: 10.1371/journal.pone.0164918 (PMC5081199; doi:10.1371/journal.pone.0164918)
Supplement: S1 Table — (DOCX) [file pone.0164918.s004.docx]

Supplementary Table 1. Characteristics of rats

| Rat | Age（month） | Sex |
| --- | --- | --- |
| 1 | 1 | F |
| 2 | 1 | F |
| 3 | 1 | F |
| 4 | 1 | F |
| 5 | 1 | M |
| 6 | 1 | M |
| 7 | 1 | M |
| 8 | 1 | M |

ASCs: Adipose-derived stem cells, SMCs: smooth muscle cells M: male, F: female
